# THE IMPORTANCE OF BRAZILIAN SOCIETY OF METABOLIC AND BARIATRIC SURGERY AND ITS INTERACTION WITH THE XXI WORLD CONGRESS OF IFSO IN BRAZIL

**DOI:** 10.1590/0102-6720201600S10001

**Published:** 2016

**Authors:** Josemberg Marins CAMPOS, Almino Cardoso RAMOS, Ricardo COHEN

**Affiliations:** 1 Presidente da Sociedade Brasileira de Cirurgia Bariátrica e Metabólica; 2 Ex-Presidente da Sociedade Brasileira de Cirurgia Bariátrica e Metabólica

From the first cases performed in 1974 and the growing interest aroused in surgeons in 1996 was founded the Brazilian Society for Bariatric Surgery, being in the same year associated with IFSO (International Federation for the Surgery of Obesity and Metabolic Disorders) which enabled the performance the 7^th^ World Congress and IFSO First World Bariatric Surgery Congress in Brazil in 2002 in São Paulo. In 2006, based on the increasing importance of metabolic surgery in the medical community it was one of the first societies to enter the name "metabolic" in its name, becoming the Brazilian Society for Bariatric and Metabolic Surgery - SBCBM. The founders followed the example of other countries, creating a society dedicated to bring together correlated professionals of the different areas, encouraging professional and technical development of the specialty.

SBCBM is one of the 17 National Societies of the Latin American Chapter of IFSO (IFSO-LAC) and has encouraged Brazilian members to actively participate in scientific events held on this continent. The IFSO-LAC Board has been working intensively, maintaining frequent communication between the partners, which has resulted in greater integration of Latin American professionals involved in bariatric and metabolic surgery. In recent years, the SBCBM is seeking greater integration with other societies of IFSO-LAC, bringing Latin American events to Brazil and supporting other studies in several countries of the continent, in order to promote regional growth and strengthening of bariatric and metabolic surgery.

In 2003, due to the increasing number of operations and, also, dedicated professionals to the area and in recognition of the need to involve multidisciplinary teams in preparation and monitoring programs of bariatric operations, SBCBM organized the creation of the Associated Specialties Commission (COESA). Every year, the society has promoted scientific and academic events to deepen the debate on obesity and bariatric surgery, addressing various topics. The First Brazilian Bariatric Surgery Congress took place in 1998, and in 2017 will be promoted the eighteenth edition in Florianopólis, SC, Brazil.

Brazil can be an example, due he is the second country in the world in number of bariatric operations, with more than 95,000 operations per year, behind only the United States. The growth in number of operations in the last ten years was 300% and the risk of bariatric procedures today is the equivalent of an abdominal surgery intermediate size.

With the increasing number of operations, SBCBM also increased, and currently has over 1700 members, among surgeons and associated specialties (endocrinologists, cardiologists, physical trainers, plastic surgeons, physiotherapists, nurses, dentists, speech therapists, nutritionists, nutritionists, and psychiatrist and psychologist). It has representatives throughout the country through the chapters and police stations. As there are controversies and controversial issues, there was the need to create rules and guidelines always looking for safer operation and better results. Officially the Practice Area in Bariatric Surgery founded in early 2015, after an important work in group with Brazilian College of Surgeons (CBC), the Brazilian College of Digestive Surgery (CBCD), the Federal Council of Medicine (CFM) and the Brazilian Medical Association (AMB), represented another important step for the Brazilian bariatric community.

In recent years, the SBCBM began the publication of articles on different topics, showing the official position of the organization, thus giving more support to its members. Published guidelines for emergency care in bariatric surgery, for revisional surgery and established the score for metabolic surgery recommendation. At the congress of IFSO in Rio de Janeiro, the symposium will focus on SBCBM bariatric surgery in adolescents. Considering the recent changes in the Brazilian legislation on bariatric surgery in adolescents, worth deepen a little the discussion on the topic to facilitate medical decision in favor of obese patients.

As in adults, obesity in adolescence - transition period between childhood and adulthood from 10 to 19 years according to the World Health Organization - has become in recent years more frequent, causing severe health consequences the younger (hypertension, dyslipidemia, diabetes, fatty liver disease, sleep apnea, psychosocial complications and comorbidities). According to the Brazilian Association for Studies of Obesity and Metabolic Syndrome - ABESO, an average of 20.72% of adolescents are overweight in Brazil.

Obesity during adolescence is worrisome, because individuals are in the physical, mental, emotional, sexual and social development period. Thus, treatment of obesity in this phase is challenging due to the psychological impact of obesity mainly meant for this age group affecting the self-esteem and quality of life. The surgeon and his team need to have specific knowledge to choose the best moment for the operation and the most appropriate treatment method. The choice of medical treatment does not always have satisfactory results, since bariatric surgery is a treatment option that has proven in adults. It is important to remember that the indicators for obesity in this age group indicate a significant increase in obesity levels since young people are increasingly sedentary and worst eating habits.

In Brazil, bariatric surgery was not indicated for adolescents; over the years, there have been changes in the parameters of the Federal Council of Medicine - CFM in relation to this group; young people between 16 and 18 years can do the operation, provided that the cost/benefit ratio is well analyzed; it must be added pediatric care with multidisciplinary team and ensured that the epiphyseal cartilage growth are consolidated. Also, in the public system the last order of the Ministry of Health of Brazil, in its comprehensive treatment program for obesity, included surgical indication for morbid obesity from 16 years. These new resolutions generated demand for the SBCBM seeking a way to study the subject to answer doubts and questions such as: What is the best treatment? What is the best technique? Surgery is harmful to psychological and physical development of adolescents?

So this year, the SBCBM conducted a systematic review, which resulted in 42 studies that comprised a total of 3488 patients, most women; the method of choice in most studies was the Adjustable Gastric Banding (BGA) since this technique does not modify the digestive system of these patients, still in development. Most of the studies mentioned that most individuals should undergo conservative treatment, and only after no satisfactory weight loss, there is indication for bariatric surgery.

The main goal of SBCBM is to encourage the treatment of obesity in adolescents, starting with clinical treatment. In cases of failure, patients should be referred to a multidisciplinary team duly trained for proper evaluation.

Thus, the SBCBM symposium during the 2016 IFSO Congress this issue will be addressed in detailed mode. The symposium title is Bariatric Surgery Metabolic in Teens - Why, when and how? dissecting the problem. Among other topics, beyond questions and answers on the subject, will be addressed: Why, when and how to make the operation? When the patient is too young? What's the procedure? Ethical standards and right connected to bariatric surgery in adolescents; Psychological impact; There is place for bands? SBCBM position on bariatric surgery in adolescents; Study of adolescent morbid obesity (AMOS - Morbid Obese Adolescent Study). The aim is to discuss controversial aspects of this issue and improve the quality of care for this patient group.

Finally, the Board is pleased to invite the global bariatric community to participate in this discussion, contributing to the theme consolidation in our society.

